# Age-Related Changes in Caudate Glucose Metabolism: Insights from Normative Modeling Study in Healthy Subjects

**DOI:** 10.3390/metabo15020067

**Published:** 2025-01-22

**Authors:** Zijing Zhang, Yuchen Li, Qi Xia, Qing Yu, Luqing Wei, Guo-Rong Wu

**Affiliations:** 1 Key Laboratory of Cognition and Personality, Faculty of Psychology, Southwest University, Chongqing 400715, China; zhangzijing@jxnu.edu.cn (Z.Z.); l20000522@email.swu.edu.cn (Y.L.); ysc8398@email.swu.edu.cn (Q.X.); yq8539562@email.swu.edu.cn (Q.Y.); 2 School of Psychology, Jiangxi Normal University, Nanchang 330022, China

**Keywords:** aging, striatal glucose metabolism, caudate, normative modeling

## Abstract

Background: As the global population ages, the prevalence of neurodegenerative conditions, such as Alzheimer’s disease (AD), Parkinson’s disease (PD), dementia with Lewy bodies, and frontotemporal dementia, continues to rise. Understanding the impact of aging on striatal glucose metabolism is pivotal in identifying potential biomarkers for the early detection of these disorders. Methods: We investigated age-related changes in striatal glucose metabolism using both region of interest (ROI)-based and voxel-wise correlation analyses. Additionally, we employed a normative modeling approach to establish age-related metabolic trajectories and assess individual deviations from these normative patterns. In vivo cerebral glucose metabolism was quantified using a molecular neuroimaging technique, ^18^F-FDG PET. Results: Our results revealed significant negative correlations between age and glucose metabolism in the bilateral caudate. Furthermore, the normative modeling demonstrated a clear, progressive decline in caudate metabolism with advancing age, and the most pronounced reductions were observed in older individuals. Conclusions: These findings suggest that metabolic reductions in the caudate may serve as a sensitive biomarker for normal aging and offer valuable insights into the early stages of neurodegenerative diseases. Moreover, by establishing age-specific reference values for caudate glucose metabolism, the normative model provides a framework for detecting deviations from expected metabolic patterns, which may facilitate the early identification of metabolic alterations that could precede clinical symptoms of neurodegenerative processes.

## 1. Introduction

The human brain, despite accounting for only 2% of total body weight, consumes a remarkable 20% of the body’s resting metabolic energy, primarily through the oxidative metabolism of glucose [1]. A significant portion of glucose metabolism sustains the brain’s functional resting state [2]. With aging, however, there is a marked reduction in cerebral glucose metabolism, which has been linked to cognitive decline and the progression of neurodegenerative conditions [3]. These observations have sparked significant interest in understanding how metabolic activity in specific brain regions changes over time.

Fluorine-18-labeled fluorodeoxyglucose positron emission tomography (^18^F-FDG PET) has emerged as a critical tool for in vivo detection of cerebral glucose metabolism. As a radiolabeled glucose analog, ^18^F-FDG is transported into cells via glucose transporters and phosphorylated by hexokinase to form ^18^F-FDG-6-phosphate. Unlike glucose, ^18^F-FDG-6-phosphate is unable to proceed through further metabolic pathways, leading to its accumulation in metabolically active tissues. This accumulation reflects regional glucose consumption and serves as a marker of brain activity. Leveraging this advanced molecular neuroimaging technique, numerous studies have shown a progressive decrease in cerebral ^18^F-FDG uptake with normal aging, particularly in cortical areas of the frontal, temporal, and parietal lobes [4,5,6,7]. Notably, age-related hypometabolism has been observed in anterior cortical regions, including the medial frontal regions and anterior cingulate gyrus, which are critical for higher-order cognitive processes such as planning, attention regulation, emotional processing, working memory, and inhibitory control [6,7]. In neurodegenerative diseases such as Alzheimer’s and Parkinson’s, ^18^F-FDG uptake also progressively decreases primarily in the frontal and cingulate cortices [8,9]. Furthermore, changes in ^18^F-FDG metabolism may occur years before the onset of symptoms, frequently preceding detectable structural alterations [10,11]. These findings suggest that metabolic declines in the fronto-cingulate areas are closely associated with cognitive deterioration and increased susceptibility to neurodegenerative processes in older adults [12,13]. Despite these advances, few studies have specifically explored the age-related changes in glucose metabolism within the striatum, a subcortical region integral to motor control, reward processing, and various higher-order cognitive functions (e.g., decision-making, cognitive control, and learning) [14,15].

The striatum, as a major component of the basal ganglia, interacts with cortical regions, such as the prefrontal cortex, to regulate voluntary movement and higher-order cognitive processes [16,17]. In age-related neurodegenerative diseases like Parkinson’s disease (PD), the striatum undergoes pathological changes, primarily due to the progressive degeneration of dopaminergic neurons [18,19]. Dopamine depletion within the striatum impairs its ability to regulate motor control, leading to hallmark symptoms such as bradykinesia, rigidity, and tremors [20]. Beyond motor deficits, reduced dopamine levels in the striatum are linked to cognitive impairments, particularly in executive functions (e.g., working memory and cognitive flexibility) [21]. These cognitive deficits can severely affect daily functioning and quality of life in PD patients. While Alzheimer’s disease primarily targets the hippocampus and cortical regions, striatal dysfunction also manifests in the later stages, contributing to both motor deficits and cognitive decline [22,23,24]. Striatal impairments in AD exacerbate dementia symptoms, including attention deficits, difficulties in planning, and impaired task-switching [21,24]. Importantly, the caudate nucleus, a critical structure within the striatum, receives inputs from various cortical areas, including the dorsolateral prefrontal cortex (dPFC) and the orbitofrontal cortex (OFC), and plays an essential role in both motor and cognitive functions, particularly in executive processes such as working memory, decision-making, and planning [25,26]. These cognitive functions are more susceptible to age-related decline, and abnormalities in the caudate have been linked to cognitive impairments, especially in conditions like PD and AD. In contrast, the putamen, which primarily integrates input from motor-related cortical regions such as the primary motor cortex, premotor cortex, and supplementary motor area, is more involved in motor functions and habitual movement patterns [25,26]. These motor functions tend to be less sensitive to early aging compared to cognitive functions mediated by the caudate. Overall, given the striatum’s critical role in motor and cognitive processes, as well as its involvement in the pathophysiology of neurodegenerative disease, investigating the impact of aging on striatal metabolism is crucial for informing strategies aimed at preserving striatal integrity and mitigating the cognitive and motor deficits commonly observed in these neurodegenerative conditions.

In this study, we aimed to explore the effects of aging on striatal glucose metabolism both at the region of interest (ROI) and voxel-wise levels. Specifically, we sought to determine how age influences glucose metabolism across different striatal subregions. Moreover, we employed a normative modeling approach to establish age-related metabolic trajectories and assess individual deviations from these normative patterns [27]. Normative modeling is an emerging statistical methodology that has shown promise in tracking disease progression in mild cognitive impairment (MCI) and Alzheimer’s disease (AD) at the individual level [28]. Like growth charts used in pediatric medicine, this approach leverages advanced statistical techniques (e.g., Gaussian modeling and machine learning) to map variation across a cohort in terms of percentiles, enabling the comparison of individual data with population-based norms [27]. By quantifying deviations from the normative range (via Z-scores), we can detect subtle, individualized changes in metabolic patterns that may be overlooked by traditional group-based comparisons. Unlike methods focused on group averages, normative modeling offers a more personalized and sensitive framework for identifying early deviations in metabolism. This is especially important in the context of aging, where neurodegenerative processes may begin long before clinical symptoms manifest [10,29]. By applying this method, our study provides deeper insights into how normal aging affects striatal metabolism and helps identify early biomarkers that may signal the onset of neurodegenerative processes.

## 2. Materials and Methods

### 2.1. Participants

The study population comprised 116 healthy individuals (39 females and 77 males, aged 20 to 81), recruited from a cross-sectional, publicly accessible Chinese-specific brain PET template study [30], with the dataset available at https://www.nitrc.org/projects/cnpet/ (accessed on 11 November 2024). All participants were free of AD, PD, diabetes, psychiatric conditions, and other neurological abnormalities, as determined by diagnostic records from PET/CT scan, blood tests, and behavior assessments. Informed consent was obtained from all participants, and ethical approval was obtained from the institutional review board of the Department of Nuclear Medicine, the First Affiliated Hospital of Dalian Medical University. The demographic information is presented in Table 1.

### 2.2. Data Acquisition and Preprocessing

PET images were obtained using a Biograph 64 PET/CT scanner (SIEMENS, Germany), with a slice thickness of 1.5 mm, an image matrix size of 336 × 336 × 110, and a pixel size of 1.01821 mm. Participants fasted for 4–6 h before receiving an intravenous injection of 0.2 mCi/kg [^18^F]-FDG, followed by a 60 min rest period. A 3 min static brain PET/CT scan was then conducted. Image reconstruction was performed using the Ordered Subset Expectation Maximization method, incorporating corrections for attenuation, scattering, stopping time, and scan normalization. Finally, static PET images were generated in SUV (standardized uptake value) units, reflecting the ratio of radioactivity concentration to the injected [^18^F]-FDG dose and the body weight.

All PET images were spatially normalized into the Chinese2020 space [31], using the Chinese brain PET template as the reference [30]. This normalization was implemented in Statistical Parametric Mapping 12 (SPM12, Wellcome Department of Imaging Neuroscience, Institute of Neurology, London, UK), and the corresponding MATLAB code is available for download at https://www.nitrc.org/projects/cnpet/ (accessed on 11 November 2024). Given potential variations in voxel intensity ranges across subjects due to differences in the acquisition procedures, intensity normalization was performed to standardize the intensity values. We adopted a widely used method for intensity normalization, where each image intensity was divided by the mean value of the voxels within the 40–90% range of the maximum brain voxel intensity.

### 2.3. ROI Correlation Analysis

Pearson correlation analyses were conducted to examine the relationship between striatal metabolism and age at the ROI level. The striatum was defined using the Automated Anatomical Labeling (AAL, https://www.gin.cnrs.fr/en/tools/aal/ (accessed on 11 November 2024)) Atlas, encompassing four areas: the bilateral caudate and putamen. Notably, given that the AAL template is in MNI space, the original AAL template was transformed into Chinese2020 space before conducting ROI analysis. Statistical significance was set at p<0.05, with correction for multiple comparisons using the Bonferroni method (i.e., p<0.05/4). To further explore sex-related effects on the relationship between striatal glucose metabolism and age, separate Pearson correlation analyses were conducted for males and females.

### 2.4. Voxel-Wise Correlation Analysis

To investigate the relationship between age and cerebral glucose metabolism at the voxel-wise level, a multiple regression analysis was conducted using SPM12. Individual [^18^F]-FDG uptake maps were treated as dependent variables, age was included as an independent variable, and sex and global mean value were included as the confounding covariates. The results were considered significant at a threshold of p<0.001, with voxel-wise family-wise-error (FWE) correction. The extent threshold was set to 50 voxels.

### 2.5. Normative Modeling Analysis

Normative modeling was performed using Python 3.9 and the PCNtoolkit package (version 0.20, https://pcntoolkit.readthedocs.io/en/latest/ (accessed on 11 November 2024)). Bayesian Linear Regression (BLR) with likelihood warping was employed to predict FDG uptake values based on age for each subject. The prediction for the FDG uptake y is expressed as follows:

$$y=w^{T}\varphi (x)+\varepsilon$$

where $w^{T}$ represents the estimated weight vector, and φ(x) is the basis expansion of the covariate vector x, using a cubic B-spline with five evenly spaced knots to model the nonlinear effect of age. The error term ε=η(0,β) follows a Gaussian distribution with a mean of zero and noise precision β (inverse variance). A likelihood warping approach [32] was applied to model non-Gaussian effects. This approach uses a bijective nonlinear warping function to map non-Gaussian responses into a Gaussian latent space, allowing for closed-form inference. In our study, a ‘sinarcsinsh’ warping function, which is equivalent to the SHASH distribution commonly used in generalized additive models, was employed [33,34].

Deviation scores (Z-scores) were computed for each subject, where a Z-score greater than 2 indicates an “extreme” positive deviation, and a Z-score less than −2 indicates an “extreme” negative deviation. The explained variance (EV) reflects the proportion of the variance in the true values accounted for by the predicted values. EV is sensitive to the mean fit and dependent on the model’s flexibility, with a value closer to 1 indicating better model performance. Model generalizability was assessed by using a half-split train test set (train: 58, test: 58) to ensure an equal distribution of sex and age across both sets. The normative trajectory was generated, illustrating the relationship between age and the predicted ^18^F-FDG uptake, with each subject in the test and train set plotted as a single point.

## 3. Results

### 3.1. ROI Correlation Analysis

Age-related changes in striatal glucose metabolism were observed in the bilateral caudate. Specifically, age was negatively correlated with glucose metabolism in the left ($r=-0.55,\;p_{uncorrected}<0.001$) and right caudate ($r=-0.56,\;p_{uncorrected}<0.001$). However, no significant correlation between age and glucose metabolism was found in the putamen (left: $r=0.16,\;p_{uncorrected}=0.089$; right: $r=0.21,\;p_{uncorrected}=0.024$).

To further validate these findings, we separately examined the relationship between striatal glucose metabolism and age in males and females. A significant negative correlation between age and metabolism was found in the bilateral caudate both in males (left: $r=-0.54,\;p_{uncorrected}<0.001$; right: $r=-0.56,\;p_{uncorrected}<0.001$) and females (left: $r=-0.42,\;p_{uncorrected}=0.007$; right: $r=-0.45,\;p_{uncorrected}=0.004$). However, no significant correlation was observed between age and metabolism in the putamen for either sex (male: left: $r=0.24,\;p_{uncorrected}=0.035$; right: $r=0.24,\;p_{uncorrected}=0.033;\;female:left:r=0.11,\;p_{uncorrected}=0.516;right:r=0.25,\;p_{uncorrected}=0.131$).

### 3.2. Voxel-Wise Correlation Analysis

**Figure 1:**
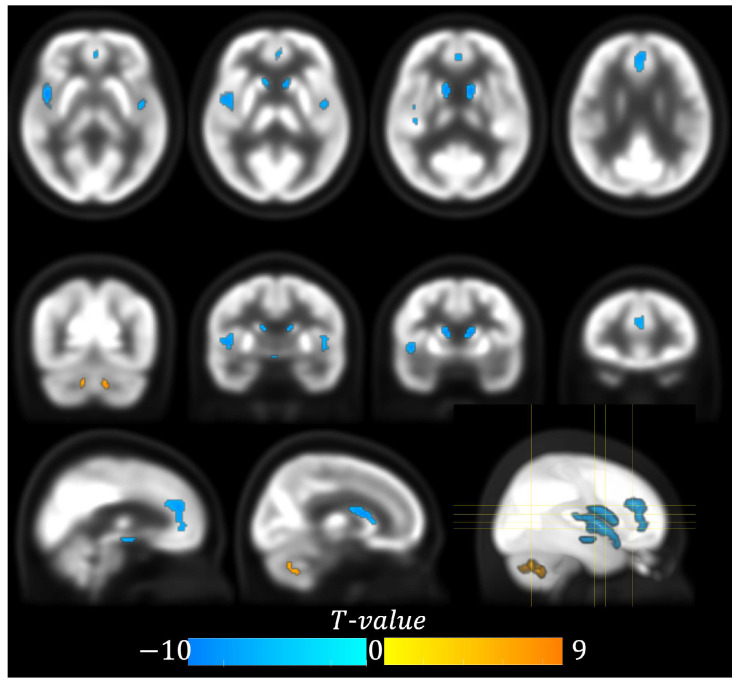
Spatial distribution of brain regions exhibiting significant correlation between age and glucose metabolism (p<0.05, FWE correction).

At the voxel-wise level, we observed a significant negative correlation between age and glucose metabolism in the bilateral caudate (peak T−value=−9.23, coordinate: x=−12, y=10, z=10, cluster size = 159 voxels), insula (peak T−value=−9.91, coordinate: x=−10, y=−60, z=−38, cluster size = 81 voxels), and anterior cingulate gyrus (peak T−value=−7.43, coordinate: x=−2, y=40, z=16, cluster size = 215 voxels) (see Table 2 and Figure 1). In contrast, the cerebellum exhibited a positive correlation with age (peak T−value=9.14, coordinate: x=−10, y=−60, z=−38, cluster size = 81 voxels). No such association was observed in the putamen.

### 3.3. Normative Modeling Analysis

**Figure 2:**
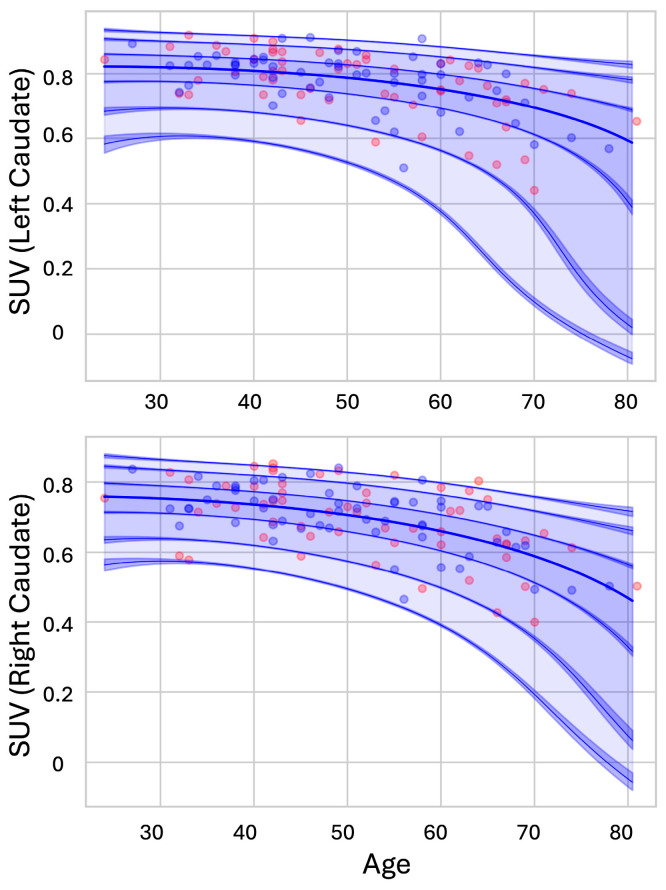
Lifespan trajectories and centiles of variation for the caudate glucose metabolism are plotted, with age on the *x*-axis and predicted standardized uptake values (SUV) on the *y*-axis. Blue circles represent the training data, while red dots denote the test data. The seven plotted fitted centile lines represent the 1st, 5th, 25th, 50th, 75th, 95th, and 99th percentiles.

The normative distribution of caudate glucose metabolism revealed a clear age-related decline, with younger individuals showing higher metabolism in the caudate, which progressively decreased with age (Figure 2). When applying the normative model to the test set, EV was 0.36 for the left caudate and 0.34 for the right caudate. The Z-scores for the left caudate ranged from −1.86 to 1.92, whereas those for the right caudate ranged from −2.26 to 2.75. Notably, four subjects exhibited extreme positive deviations (Z>2), and two subjects exhibited extreme negative deviations (Z<−2) in the right caudate (Figure 2 bottom). The Z score, or deviation score, quantifies the degree to which each subject deviates from the normative range. Such deviations can be observed in both clinical and healthy cohorts, although they are more frequent in clinical populations [28,35]. Extreme deviations observed in the right caudate may reflect the increased heterogeneity of atrophy within this region as aging progresses. Additionally, normative modeling with the putamen showed no predictive power (EV < 0), suggesting that the relationship between age and putamen glucose metabolism is less clear or less consistent compared to the caudate. This indicates that factors other than age might contribute to variations in putamen metabolism, or that the age-related metabolic decline in the putamen may not follow the same predictable pattern observed in the caudate.

## 4. Discussion

The results from both voxel-wise and ROI-based correlation analyses, along with the normative modeling, provide compelling evidence of age-related declines in caudate glucose metabolism. Specifically, significant negative correlations with age were observed in the bilateral caudate, consistent with previous research on striatal aging [36,37]. More importantly, the normative model further confirmed a clear, age-related reduction in caudate metabolism, with the most pronounced decline observed in older individuals. Based on the critical role of the caudate in motor and cognitive functions, our findings suggest that caudate metabolism decline could serve as a sensitive biomarker for both normal aging and the early stages of neurodegenerative processes.

The striatum, composed of the caudate nucleus and the putamen, is a key input structure of the basal ganglia and plays a critical role in the regulation of voluntary movement, coordination, and higher-order cognitive processes, including attention, decision-making, and working memory [17,38,39]. The striatum undergoes both structural and functional alterations that can significantly contribute to age-related cognitive and motor decline [40,41]. Marked neurobiological changes have been observed in the striatum, particularly, a progressive decline in dopamine receptor density and dopamine synthesis capacity [36,42,43]. This decline in dopaminergic function impairs the striatum’s ability to effectively regulate motor and cognitive processes, resulting in slower motor performance, difficulties in motor learning, and cognitive deficits such as reduced attention, cognitive flexibility, and decision-making ability [44,45,46]. These age-related alterations are not only hallmarks of normal aging, but are also closely linked to the early stages of neurodegenerative diseases. For example, in Parkinson’s disease, where dopaminergic neurons are progressively lost, the striatum’s capacity to manage motor and cognitive control becomes severely compromised [47,48]. Our study found age-related metabolic changes in the striatum, highlighting its crucial role in both normal aging and the progression of neurodegenerative disorders.

Notably, in our study, age-related metabolic changes were detected within the bilateral caudate. This subcortical structure, which contains several neuronal clusters functionally connected to cortical regions, is typically divided into ventral and dorsal regions [26]. The ventral caudate, through its connections with the orbitofrontal, medial prefrontal, and anterior and midcingulate cortices, is responsible for emotional and affective processes, including reward processing [49]. In contrast, the dorsal caudate, which primarily connects with the dorsolateral prefrontal cortex, plays a crucial role in cognitive and executive functions, such as spatial working memory [50]. Functional connectivity studies suggest that the head of the caudate is primarily associated with cognitive and emotional networks, while the body of the caudate is more involved in perceptual and motor control functions [51,52]. Previous studies have shown an age-related volumetric decline in the bilateral caudate [53,54], which can be linked to cognitive impairments in the elderly. For instance, caudate atrophy is associated with longer action selection times and poor action selection performance in older individuals [55]. In addition, age-related dopamine loss has been observed in the caudate [56]. Beyond normal aging, the caudate is also implicated in neurodegenerative disorders, particularly those associated with cognitive decline and age-related diseases. Notable examples include frontotemporal dementia [57,58], Parkinson’s disease [59], and Alzheimer’s disease [60], where patients exhibit both dopaminergic and cholinergic degeneration, accompanied by volumetric decline within the caudate. Our findings of age-related metabolic decline in the caudate align with similar declines observed in other cortical regions, such as the frontal, temporal, and parietal cortices, which are known to undergo metabolic changes with aging [9,61,62]. These cortical regions, particularly the medial prefrontal cortex and anterior cingulate, are responsible for executive functions and emotional regulation, and their metabolic reductions have been closely linked to cognitive decline and aging-related neurodegeneration [8,29]. Taken together, metabolic decline in the caudate observed in our study adds to the broader understanding of how both subcortical and cortical structures are affected by aging. Moreover, the current study found age-related metabolic decline in the caudate, suggesting that reductions in caudate metabolism may act as a potential biomarker for cognitive decline and neurodegeneration associated with aging [12,13].

Normative models of aging are increasingly valuable [33], given the well-documented link between aging and changes in brain tissue, which are central to cognitive decline and neurodegenerative diseases [28,61]. In addition to shedding light on normal aging processes, normative models are also becoming instrumental in identifying and characterizing brain abnormalities in clinical populations [33,63]. These models provide insights beyond traditional case–control approaches, which assume the mean is representative of the population, a premise that may not always be valid [27]. For instance, one large-scale study analyzing over 15,000 MRI scans developed a lifespan normative model of cortical thickness and subcortical volume [64]. This model revealed that individuals with internalizing disorders exhibit deviations primarily in subcortical regions, while those with psychotic disorders show deviations in both cortical and subcortical areas [64]. In another recent study, normative references derived from healthy samples were applied to an independent cohort of healthy individuals and patients with schizophrenia. The researchers found a significantly higher proportion of schizophrenia patients with infra-normal volumes in the bilateral mediodorsal, pulvinar, and medial geniculate nuclei, highlighting the power of normative models in detecting neural deviations specific to diagnostic groups [35]. Collectively, these studies demonstrate the capacity of normative modeling to capture individual variability, not only characterizing typical brain aging, but also identifying pathophysiological changes across diverse clinical populations.

To the best of our knowledge, this is the first study to employ normative models to characterize glucose uptake across the lifespan, providing a novel framework for understanding age-related metabolic changes. By examining the normative trajectories of the caudate nucleus, we were able to directly relate age to predicted glucose uptake, revealing a clear age-related decline in caudate metabolism. This decline is particularly evident in older individuals, supporting the notion that metabolic changes in the caudate may serve as a sensitive biomarker for normal aging and neurodegenerative processes. Unlike previous lifespan studies that primarily focused on volumetric changes [54,55] or regional perfusion [62,65], our study is the first to apply normative modeling to characterize metabolic trajectories of glucose uptake in the caudate. Consistent with our findings, previous research has demonstrated significant metabolic decline in the caudate with aging [62] as well as age-related reductions in perfusion within the caudate nucleus [65]. Our results further align with studies linking aging to dopamine depletion in the caudate [56]. The application of normative modeling in this study provides a unique and valuable perspective on the age-related decline in caudate metabolism, offering new insights into the early detection of cognitive and neurodegenerative changes. Moreover, the normative model we developed allows for the prediction of expected glucose metabolism values in the caudate at different ages, establishing a framework to detect deviations from normative patterns in clinical populations. This could be especially useful for the early identification and monitoring of neurodegenerative diseases.

One of the potential limitations of this study is its cross-sectional design, which only provides a snapshot of the relationship between age and caudate metabolism at a single point in time. While we observe significant age-related metabolic declines, the lack of longitudinal data means we cannot conclusively establish causal relationships or track how metabolic changes progress over time. Secondly, although our sample included healthy individuals across a broad age range, this study’s sample size may still be considered limited for generalizability, especially when subdividing the sample into specific age groups. A larger sample would strengthen the robustness of both the training and testing sets, thereby improving the model’s accuracy and precision in capturing the target phenotype. Additionally, the study population may not fully represent the diversity of the general population in terms of factors like sex, ethnicity, and comorbidities, which could influence caudate metabolism. Thirdly, our study focused exclusively on healthy individuals, so we cannot draw direct conclusions regarding the metabolic changes in clinical populations with neurodegenerative diseases, such as Alzheimer’s disease or Parkinson’s disease. To enhance generalizability and clinical relevance, future research can expand this normative modeling framework to include patients with AD and PD. While the utility of ^18^F-FDG PET for assessing glucose metabolism in neurodegenerative diseases is well-established [29], few studies have applied normative modeling to patient populations. By using normative modeling to explore glucose metabolism, we can uncover more nuanced and diverse patterns of metabolic decline within and between clinical populations, thereby addressing the heterogeneity inherent in neurobiological profiles of neurodegenerative disorders [64]. These insights could ultimately facilitate personalized healthcare and advance precision medicine [28].

## 5. Conclusions

In summary, our study provides strong evidence of age-related changes in striatal glucose metabolism, particularly within the caudate nucleus. Both ROI-based and voxel-wise correlation analyses revealed significant negative correlations between age and glucose metabolism in the bilateral caudate, further substantiated by the application of a normative modeling approach. Our results demonstrate a clear, progressive decline in caudate metabolism with advancing age, with the most pronounced reductions observed in older individuals. These findings underscore the potential of caudate metabolism as a biomarker for the early detection and monitoring of age-related cognitive and motor declines, as well as the neurodegenerative processes that accompany aging. Furthermore, by establishing age-specific reference values for caudate glucose metabolism, the normative model provides a valuable framework for detecting deviations from expected metabolic patterns, which could be crucial for identifying early metabolic alterations that precede clinical symptoms of neurodegenerative processes. In future studies, researchers can expand this model to clinical populations, such as individuals with Alzheimer’s disease or Parkinson’s disease, to better understand how age-related changes in caudate metabolism intersect with disease progression. Additionally, longitudinal studies that track caudate metabolism over time could offer deeper insights into how metabolic declines in this region contribute to the onset of neurodegenerative processes. Ultimately, these efforts could help refine early diagnostic tools and guide interventions aimed at preserving cognitive and motor function in aging populations.

## Figures and Tables

**Table 1:** Demographic information of the study sample.

Gender/Age	20–30	31–40	41–50	51–60	61–70	71–81
Male	2	14	18	18	20	5
Female	0	9	16	11	3	0

**Table 2:** The regional glucose metabolism showed a significant correlation with age (p<0.001, voxel-wise FWE correction).

Cluster	Anatomical Region	Cluster Size (#Voxel)	Peak Coordinates (x, y, z) Chinese2020 Space	Peak t-Value (*df* = 112)
1	Left Cerebellum 7b	81	−10, −60, −38	9.14
2	Right Cerebellum 9	52	8, −46, −40	7.18
3	Right Insula	303	42, 8, −4	−9.91
4	Left Caudate	159	−12, 10, 10	−9.23
5	Right Caudate	125	10, 12, 8	−8.71
6	Anterior Cingulate Gyrus	215	−2, 40, 16	−7.43
7	Left Insula	72	−42, −2, −4	−7.45

## Data Availability

The data used in this study are publicly available (https://www.nitrc.org/projects/cnpet/), accessed on 11 November 2024.
